# The role of neutrophils in vasospasm and delayed cerebral ischemia after aneurysmal subarachnoid hemorrhage: Is it time for a SAH clinical trial targeting neutrophils?

**DOI:** 10.1177/0271678X251370858

**Published:** 2025-09-08

**Authors:** William W Wroe, Hussein A Zeineddine, Spiros L Blackburn, Jaroslaw Aronowski, Devin W McBride

**Affiliations:** 1 The Vivian L. Smith Department of Neurosurgery, McGovern Medical School, The University of Texas Health Science Center at Houston, Houston, TX, USA; 2 Department of Neurology, McGovern Medical School, The University of Texas Health Science Center at Houston, Houston, TX, USA

**Keywords:** Neutrophils, subarachnoid hemorrhage, delayed cerebral ischemia, delayed neurological deficits, vasospasm, NETs

## Abstract

Aneurysmal subarachnoid hemorrhage (aSAH) is a devastating neurological disease, and one of the primary drivers of morbidity after aneurysm rupture is the phenomenon of delayed cerebral ischemia (DCI). Significant knowledge has been gained over the past two decades of the impact of neuroinflammation in DCI; and neutrophils are now believed to play a major role. There is significant human subject data showing the rise of neutrophil related inflammatory markers and neutrophil’s association with poor outcome after aSAH, but as of yet no trials involving human subjects have been done specifically targeting neutrophils. There is however a growing body of evidence in animals models that targeting neutrophils, or their byproducts such as neutrophil extracellular traps improves outcomes. This review summarizes the available evidence of neutrophil’s impact in both human subjects and animal models of aSAH and should serve as an impetuous to explore clinical trials in human subjects.

## Introduction

Aneurysmal subarachnoid hemorrhage (aSAH) accounts for 5%–10% of all strokes in the United States, affecting about 9/100,000 people/year.^1^ Delayed cerebral ischemia (DCI) is the most common cause of morbidity in patients with aSAH who survive to having their aneurysm definitively treated. DCI occurs in roughly 30% of surviving patients, usually within 3–14 days after aSAH onset, and often leads to cerebral infarction or delayed neurological deficits (DND).^2^ Historically, DCI was thought to be caused by large vessel vasospasm, often referred to as angiographic vasospasm. However, while two-thirds of aSAH patients will experience angiographic vasospasm, only about half of these patients will develop DCI. Moreover, even patients who do not develop angiographic vasospasm can suffer from DCI. Nimodipine, the only FDA-approved medication for DCI in the United States, decreases the incidence of DCI with minimal effect on angiographic vasospasm.^3^ Finally, older randomized clinical trials using endothelin receptor antagonists were effective at preventing angiographic vasospasm without relieving DCI or improving clinical outcomes.^4^ These findings have shifted the field of DCI research towards alternative explanations and contributing factors, such as microthrombi, cortical spreading ischemia, microvascular constriction, and inflammation.^3^ The effects of endothelin receptor antagonists and the relationship between angiographic vasospasm and DCI continue to be studied though with more recent trials in Japan showing both reduced vasospasm and improved clinical outcomes with clazosentan treatment,^5^ whereas a large international multicenter trial failed to show any statistically significant clinical benefit.^6^

It well-known that aSAH causes an inflammatory response,^7^ and there are several published review articles that describe a plethora of experimental and clinical evidence for inflammatory mechanisms.^8–10^ Post-SAH inflammation is not a passive response to aneurysm rupture, but rather inflammation plays a significant causative role in the development of DCI and DND in the days following aneurysm rupture.^11,12^

Neutrophils are an immune cell integral to the function of the innate immune system, and are the most abundant immune cell in humans. They are the most predominate type of granulocyte cell and have many anti-microbial and inflammatory mechanisms.^13^ With regards to aSAH, neutrophils are gaining attention in post-SAH pathophysiological events and they may even play a critical role in DCI. Of particular interest are the neutrophil extracellular traps (NETs) and the potential for neutrophils to induce microthrombi. The focus of this review is to summarize the evidence for neutrophil’s role in DCI, as well as to discuss the studies focusing on neutrophils as a therapeutic target for SAH.

## Clinical evidence of neutrophil involvement in human aSAH and DCI

### Blood makers of neutrophil activity

Over the decades, there has been a considerable number of studies examining blood collected from aSAH patients. As neutrophil counts and the ratios of neutrophils relative to other cell types are related to overall morbidity and specific pathophysiological effects after ischemic stroke^14–16^ and intracerebral hemorrhage,^17,18^ there has been a quickly expanding volume of large, and mostly retrospective, studies examining blood neutrophil counts on admission and its association with deleterious outcomes after aSAH.

Admission neutrophil count has been reported to positively associate with DCI and poor outcome. Zhang et al. retrospectively reviewed 6041 aSAH patients from four hospitals, and using a propensity matched analysis with a high neutrophil cutoff equal to 8.88 × 10^9^/L, found an increased odds of mortality in the high neutrophil group (OR 1.42, 95% CI 1.10–1.83).^19^ Work by our team measured CBCs daily in 451 aSAH patients and observed that blood neutrophil count declined from PBD 0 (13 × 10^9^/L) until PBD 5, but then neutrophil count rose from PBD 5 to 8, the last date studied. In patients who developed DCI, blood neutrophil count was significantly higher than non-DCI patients on each day; neutrophil-to-lymphocyte ratio (NLR) was only higher on PBD 2, 3, 4, and 7.^20^

NLR is thought to better reflect the state of the immune system than neutrophil count alone due to its incorporation of the balance between innate and adaptive immunity,^21^ and in stroke patients it has been shown to correlate with increasing hemorrhagic transformation,^22^ peri-hemorrhage edema,^18^ and poor outcome.^23^ In keeping with this line of reasoning, several studies have also examined the NLR based on the admission complete blood count (CBC) and have reported positive correlations between NLR and both DCI and poor outcome. However the effect size is relatively small, and cut off values for what is considered an elevated NLR varies significantly between studies (4.0–11.47).^24–30^ A recent meta-analysis by Guo et al. that included 4840 aSAH patients from 19 studies, reported that an elevated NLR was significantly associated with poor outcome after aSAH (OR 1.31, 95% CI 1.13–1.49). Of the 4840 aSAH patients, DCI was assessed in 3906 of them and they reported that NLR was also positively correlated with DCI (OR 1.32, 95% CI 1.11–1.56).^31^ Wu et al. tested for a relationship between admission NLR and CT perfusion (CTP) in 122 patients. In addition to observing a statistically significant positive correlation between NLR and DCI, there was a strong negative correlation between NLR and cerebral blood flow (CBF) and a positive correlation between NLR and mean transit time, an important variable in detecting tissue at risk from ischemic injury.^29^

Finally, two other ratios that have been reported are the neutrophil-to-albumin ratio (NAR) and the neutrophil percentage-to-albumin ratio (NPAR). A reduction in blood albumin levels is often seen as a response to acute inflammation and is associated with poor outcomes in many diseases including aSAH.^32^ Additionally, NAR and NPAR have been linked to poor outcomes in varying pathologies^33,34^ including myocardial infarction,^35^ ischemic stroke,^36^ and cardiogenic shock.^37,38^ In hopes of finding a better prognostic indicator then NLR, several groups have evaluated the NAR and NPAR and its correlation with DCI and poor outcome after aSAH.^39–42^ Zhang et al. retrospectively reviewed 3173 aSAH patients and observed a hazard ratio of 1.78 (95% CI 1.49–2.12) between NAR levels and long-term mortality.^42^ Work by Zhang et al. also reported a strong association between NAR and DCI (NAR equal to 0.350 in DCI group vs 0.240 in non-DCI group, *p* < 0.01),^40^ as well as a positive correlation between NAR and poor outcome at 3 months (GOS score 4–5: 0.231, GOS score 1–3: 0.349, *p* < 0.001).^39^

### Cerebrospinal fluid makers of neutrophil activity

Numerous studies have looked at cytokine analysis after aSAH both in the cerebrospinal fluid (CSF) and blood.^8,9,43–46^ Since SAH is a devastating acute event (i.e. rupture of an aneurysm) and also has a prolonged period of aneurysm formation and growth prior to SAH, there is a large amount of cytokines and chemokines which have been identified as contributing factors to SAH outcomes. However, the majority of these inflammatory factors are non-specific markers of inflammation and can trigger the activation or recruitment of various cell types. Many of these factors have been reviewed previously, and will not be the focus of this review.^47^

One specific family of inflammatory factors is the cell adhesion molecules (CAM) which are important in trafficking of leukocytes, including neutrophils.^48^ The first study to evaluate the presence of CAMs in aSAH patients was by Polin et al., in which the authors compared the CSF levels of E-selectin, ICAM-1, VCAM-1, and L-selectin. Their study of 17 aSAH patients reported significantly higher levels of CAMs in the CSF of aSAH patients compared to those of control patients.^49^ In a similar study, Kaynar et al. reported that ICAM-1 and VCAM-1 were significantly higher in both the CSF and serum after aSAH, and that the levels of these CAMs continued to rise over time from PBD 0–3 through PBD 7.^50^

Other cytokines involved in neutrophil recruitment and migration have also been reported following aSAH. Osuka et al. found that IL-8, ENA-78, C5a, and CXCL1 levels were all increased in patient’s CSF after aneurysmal SAH when compared to control CSF.^51^ Coulibaly et al. showed that IL-17, which is known to recruit neutrophils, was increased in human CSF after SAH and correlated with poor outcome. No DCI related outcomes were reported.^52^

More direct evidence of neutrophil involvement in DCI after aSAH came from work by Provencio et al. which examined neutrophil count on PBD 3 in 70 aSAH patients. The authors found that the percentage of CSF neutrophils on PBD 3 was significantly higher in the 25 patients that developed vasospasm compared to that of the 45 patients who did not develop vasospasm (76% vs 52%, *p* = 0.009).^53^ In a study by Osuka et al., two markers of neutrophil activity, myeloperoxidase (MPO) and neutrophil gelatinase-associated lipocalin, were elevated in CSF collected from aSAH patients.^51^ This evidence of increased neutrophil activity in the CSF is also supported by Moraes et al. who reported that the percentage of CD69^+^ (a marker of neutrophil activation) neutrophils was significantly higher in the CSF than in the serum of aSAH patients.^54^ One limitation shared by these studies is that the sample sizes were small to modest. While the evidence suggests that the CSF of aSAH patients contains a significant amount of activated neutrophils, whether or not CSF neutrophils are directly involved in DCI after aSAH remains unknown.

### Neutrophil extracellular traps

Neutrophils have a variety of mechanisms they can perform. A particular event, termed neutrophil extracellular traps (NETs), is the release of DNA, cytosolic granules, and other proteins to form an extra-cellular web-like structure. NETs were initially discovered in 2004 as a bacterial trapping mechanism,^55^ however their role in coagulation and thrombosis was elucidated in 2010.^56^ Since then, NETs have been shown to have pathological involvement in numerous vascular diseases.

**Figure 1.**
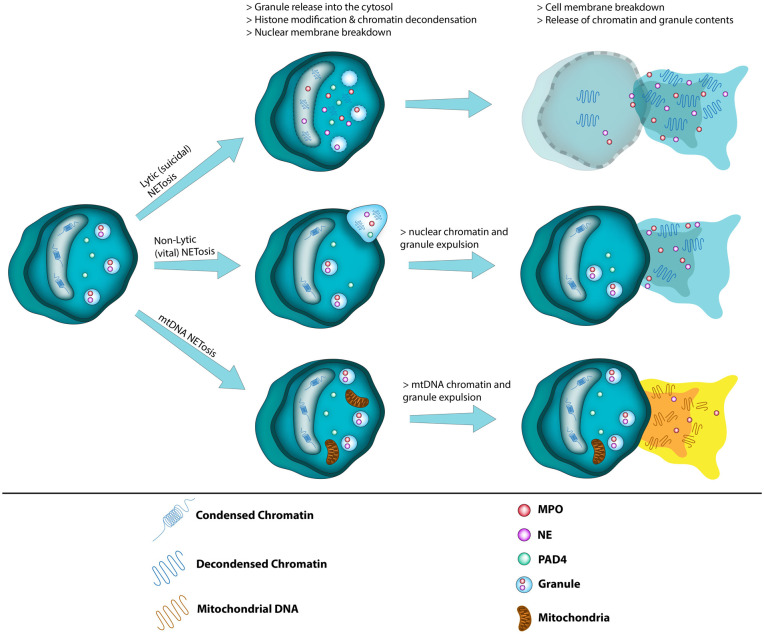
The three pathways of NETosis.

**Figure 2.**
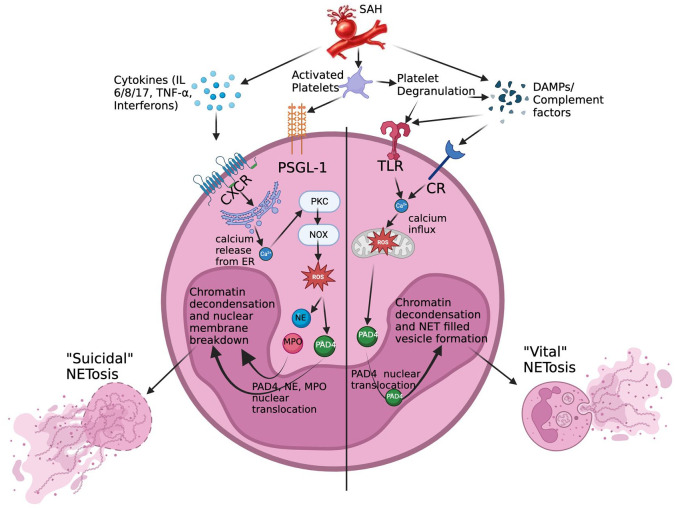
Known receptors and intra-cellular mechanisms in suicidal and vital NETosis.

Many of the underlying mechanisms of NETosis are still being uncovered, however there are currently two main pathways that neutrophils use to excrete NETs.^57^ The first is the lytic or suicidal pathway that involves cell death and lysis of the cell membrane as part of NET excretion.^58^ In the second pathway called non-lytic or vital NETosis, the neutrophil retains its viability after excretion of NETs.^59^ Figure 1 depicts these two pathways as well as a third mechanism of NETosis involving only mitochondrial DNA excretion.^60^ Whether it is the lytic or non-lytic pathway, that is, dominate in SAH induced NETosis is not known at this time. Figure 2 shows the receptors and some of the intracellular mechanisms known to be involved in these two pathways following SAH. The relevance of mitochondrial NETosis to SAH is unclear as there have only been a limited number of in-vitro experiments studying this pathway of NETosis.

Recently, NETs have become a new area of focus as contributing to post-SAH secondary injury and NETs may even be a potential cause of DCI.^61^ As such, several research groups have begun looking for evidence of NET involvement in aSAH patients. There are many different potential markers of NETosis as shown in Table 1, and each method has its own advantages and drawbacks.^62–64^ The various research groups in this field have used different combinations of these markers to quantify NETosis.

**Table 1.** Laboratory markers of NETosis.

Marker	Description	Pro/cons	SAH studies
Citrullinated histones (e.g. citH3, citH2b)	During the rapid intracellular DNA decondensation during NETosis, the histones attached to DNA are citrullinated by PAD4	Only PAD4-dependent NETosis identified	Animal: ^65,67,68,71,83,106,107^ Human: ^68–71^
dsDNA	Released by neutrophils during NETosis. Broken down by DNAse	Not specific to cell-death derived from NETosis	Animal: None Human: ^65^
cfDNA	Consists of fragments of dsDNA and single stranded DNA originating from cells undergoing cell death including those neutrophils undergoing NETosis. Broken down by DNAse	Not specific to cell-death derived from NETosis	Animal: None Human: ^70^
Extracellular histones (e.g. H2A, H2B)	Released by neutrophils during NETosis. The most abundant proteins in NETs	Not specific to cell-death derived from NETosis	Never studied in SAH. Studied in COVID^115^
PAD4	An enzyme involved in the citrullination of histones, one of the key steps in PAD4-dependent NETosis	Not sensitive to PAD4-indepdnent NETosis pathways	Animal: None Human: ^70^
NE	A proteinase enzyme stored in neutrophil granules. Released by neutrophil during degranulation and NETosis	Its presence is not specific to NETosis derived degranulation	Animal: ^67,68,106,107^ Human: ^71^
MPO	A peroxidase enzyme stored in neutrophil granules. Released by neutrophil during degranulation and NETosis	Its presence is not specific NETosis derived degranulation	Animal: ^67,107^ Human: None
MPO–DNA complexes	Complexes formed by the neutrophil derived protein MPO and the NETosis remnant cf-DNA suggest the presence of NETs	High specificity to NETosis	Animal: None Human: ^69^
NE–DNA complexes	Complexes formed by the neutrophil derived protein NE and the NETosis remnant cf-DNA suggest the presence of NETs	High specificity to NETosis	Never studied in SAH. Studied in vasculitis^64^
H3Cit–DNA complexes	Complexes formed by the neutrophil derived protein MPO and the NETosis remnant citH3 suggest the presence of NETs	High specificity to NETosis, Only PAD4-dependent NETosis identified	Never studied in SAH. Studied in myeloma^116^

cfDNA: cell-free DNA; dsDNA: double stranded DNA; H3Cit: citrullinated histone H3; MPO: myeloperoxidase; NE: neutrophil elastase; PAD4: protein arginine deiminase 4.

Früh et al. measured the levels of double stranded DNA (dsDNA) in the peripheral blood and CSF of 20 aSAH patients on PBD 1, 2, 7, and 14. dsDNA, as a surrogate maker for NETs, peaked on PBD 7 in both blood and CSF, and was significantly higher on all days compared to those of control patients.^65^ Wu et al. evaluated blood levels of triggering receptor expressed on myeloid cells 1 (TREM1) in aSAH patients. TREM1, a transmembrane protein expressed on neutrophils, has been linked as an upstream factor that potentiates the release of NETs from neutrophils.^66^ On admission, blood TREM1 levels were significantly elevated in the 12 aSAH patients versus the healthy controls, and positively correlated with Hunt Hess (HH) grade.^67^ Zeng et al., using citrullinated histone H3 (CitH3) as a surrogate marker of NETs, found that blood levels of CitH3 were significantly elevated in their 10 aSAH patients versus controls and that CitH3 levels had a positive correlation with HH grade.^68^ While these studies suggest NETs are elevated after aSAH, the studies all contained a small sample size and none of them tested for a correlation with vasospasm or DCI.

In 2022, Witsch et al. was the first study to examine the potential correlation between NETs and DCI. The authors measured the levels of MPO–DNA complexes in the blood from 78 aSAH patients on PBD 0 and 4, and observed that admission MPO–DNA complex levels were similar between patients who experienced DCI and those that did not. Interestingly, on PBD 4 there was a significant reduction in the MPO–DNA complex levels in the patients who experienced clinical vasospasm, defined as either the presence of DND or abnormal transcranial dopplers. The authors suggested that the reason for the reduction of these complexes in clinical vasospasm patients was possibly due to NET-driven thrombus formation that binds the MPO–DNA complexes, thereby removing the complexes from the blood.^69^ In a follow up study of the same patients, Hendrix et al. measured several other NET biomarkers (CitH3, PAD4, cell-free DNA, and DNAase I activity) and found similar levels on PBD 0 between the symptomatic and non-symptomatic vasospasm groups. However, the DCI group displayed a statistically significant decline in DNase I activity from PBD 0 to 4, whereas the non-DCI group did not, suggesting that the reduced clearance of NETs due to reduced DNase I activity is involved in development of DCI.^70^

A study by our own group evaluated 127 aSAH patients, 40 of whom developed DCI. While all aSAH patients had an elevation in blood levels of neutrophil elastase and CitH3, the level of neutrophil elastase was significantly higher in DCI patients on PBD 2, 7, and 10 compared to those of the non-DCI group. Interestingly, CitH3 levels were only significantly higher in DCI patients on PBD 10. The delayed increase in CitH3 levels may be due to impaired degradation of NETs. In addition to blood markers of NETs, brain tissue was collected from two aSAH patients and evaluated for intravascular NETs. We did not observe NETs in the brain tissue from the patient who did not develop DCI, but in the aSAH patient that developed DCI, NETs were observed occluding the brain blood vessels. This is the first reported case of NETs being seen in the human central nervous system (CNS) vasculature after aSAH.^71^

## Animal model evidence of neutrophil involvement in aSAH and DCI

Although there are various animal models of SAH,^72,73^ a strong inflammatory response with features similar to that seen in human aSAH is consistently reported. A recent systemic review by Devlin et al. examined publications from 2015 to 2021 reporting on cytokines in SAH in both humans and animal models,^47^ and while the growing clinical evidence of neutrophil involvement in DCI is often indirect and involves only observational data, there is extensive experimental evidence illuminating the role of neutrophils in post-SAH pathophysiology.

Several studies have observed increased neutrophil interaction with brain vascular endothelial cells early (10 min–24 h) following SAH.^74–76^ Yang et al. examined this phenomenon using in vivo microscopy of the microcirculation and reported that from 0 to 3 h post-SAH, there was an immediate rise in adherent leukocytes to pial venules, which persisted throughout the experiment, but these vessels did not display vasoconstriction. However, the authors reported that pial arterioles displayed significant vasoconstriction, but they did not analyze neutrophil adhesion to these vessels.^76^ Use of fasudil, a protein kinase inhibitor used clinically in Japan, has been reported to inhibit neutrophil infiltration after SAH in canines.^77^ But since fasudil can prevent cerebral vasospasm,^78,79^ and has other mechanisms of action, it is unknown whether fasudil directly inhibits neutrophil infiltration or if this is an indirect effect caused by fasudil’s other mechanisms of action.

### Neutrophil depletion

While studies directly observing neutrophil adherence to endothelial cells and infiltration into the brain are interesting, especially viewing neutrophils interacting with vessels experiencing vasospasm, the question remains if the neutrophils are only responding to the cytokine storm associated with SAH. To determine if neutrophils really are a key cell type in the pathophysiological events following SAH, neutrophil depletion studies offer great insight. Work by Provencio et al. was not only the first study of neutrophil depletion but also the largest body of work studying the role of neutrophils in SAH. In their earliest study, Provencio et al. utilized a Ly6G/C antibody and observed a significant reduction in vasospasm of the middle cerebral artery (MCA) on PBD 1 and 6. More intriguingly, DND, evaluated via neurobehavioral testing, was lower in the group treated with the Ly6G/C antibody than that of the control antibody SAH group.^80^ While the antibody used in their initial study targeted neutrophils and monocytes, Provencio et al. followed up with a second study using an antibody which is more specific to neutrophils.^81^ The authors evaluated vasospasm on PBD 6 in mice treated with the Ly6G antibody either 1 day before, 12 h after, 1 day after, or 3 days after SAH. Antibody administration on day 3 post-SAH was the only treatment regimen which showed a significant reduction in PBD 6 MCA diameter. Using this day 3 treatment group, the authors then evaluated spatial memory using the Barnes maze test from PBD 5 to 9 and the Morris water maze from PBD 30 to 34. In both neurobehavioral tests, the mice receiving the neutrophil depletion treatment had neurobehavioral performance indistinguishable from the sham group and which was significantly improved compared to the control antibody SAH group. The neurobehavioral improvement was correlated with electrophysiological differences in the NMDA receptors of the hippocampus. Important to note is that minimal neutrophil invasion was seen in the hippocampus for all groups, but the control antibody SAH group displayed significantly more microglial activation suggesting a link between neutrophils and microglial brain inflammation, that is, not dependent on direct neutrophil invasion of the brain parenchyma.^81^

Work by Friedrich et al. investigated neutrophil invasion and endothelial damage after SAH in rats using three treatments: vinblastine, rabbit anti-rat PMN serum (neutrophil depletion), and pyrrolidine dithiocarbamate. Friedrich et al. observed that neutrophils attached to the endothelium and invaded the CNS within 10 min after SAH, and that neutrophil depletion reduced endothelium loss.^74^ The authors did not examine any time point past 24 h, nor did they analyze vasospasm or neurobehavior.

The effect of neutrophils on cerebral perfusion after SAH was examined by Neulen et al. Using a Ly6G antibody 12 h prior to SAH induction, the authors observed that neutropenia led to a significant increase in cortical perfusion 3 h after SAH, compared to that of the control antibody, without a difference in cerebral perfusion pressure. While the authors also examined cortical perfusion at 15 min and 24 h post-SAH, no significant differences were observed for these timepoints. Finally, they also reported that neutropenia reduced the mRNA expression of three inflammatory markers: IL1β, iNOS, and TNF-α.^82^

Work by our group also supports the role of neutrophils in cerebral hypoperfusion after SAH. Neutrophil depletion led to a significant increase in CBF after SAH which was correlated with reduced cerebral infarction and improved neurological function. Neutrophil depletion using an atni-Ly6G antibody also prevented the development of DND, supporting the work by Provencio et al. which reports that neutrophils are key contributors to DCI.^60^

Finally, Zeng et al. and Hao et al. also reported that neutrophil depletion improves neurological outcomes at 24 h after SAH, suggesting a role for neutrophils in early brain injury (EBI).^68,83^

While experimental evidence is compelling for the therapeutic benefits provided by neutrophil depletion, unfortunately it is not a realistic therapeutic option due to the profound immunosuppression neutrophil depletion would cause in this patient population known to be vulnerable to infection.^84^ This necessitates more focused disruptors of the neutrophil functions more specific to vasospasm and DCI.

### Neutrophil inhibition

As neutrophils have been established in the pathogenesis of SAH via endothelial cell interaction and infiltration, microglial activation, and blood vessel occlusion, therapeutic targets disrupting these effects are now being studied. To date, several studies have looked at novel ways to inhibit neutrophils during SAH. These potential mechanisms of inhibiting neutrophils are presented in the following paragraphs.

Aneurysm rupture and subsequent SAH causes a cytokine storm which activates neutrophils, among other cell types. Some of the cytokines and chemokines released following SAH serve as chemoattractants to guide immune cells towards the site of injury. One such chemoattractant for neutrophils that has been shown to be elevated after experimental SAH is leukotriene B4 (LTB4).^85^ Using a rat model of SAH, Ye et al. evaluated an inhibitor of LTA4 hydrolase (which converts LTA4–LTB4) injected directly into the ventricles 10 min after SAH. In mice which received the LTA4 hydrolase inhibitor, the authors found a significant reduction in LTB4 levels in the cortex as well as a concomitant reduction in neutrophil infiltration into the CNS, reduced brain levels of pro-inflammatory cytokines and oxidative stress markers, and increased neuronal survival. Brain edema was also significantly lower, and neurological function was improved at 24 h for the LTA4 hydrolase group. However, at 72 h post-SAH, the very beginning of the DCI window, the treatment effects were no longer apparent.^86^

Programmed death-1 (PD-1) is an inhibitory immune check-point receptor expressed on activated immune cells and binding of its ligand reduces the level of cellular immune response. It has been a common target in oncology, but recent animal studies suggest it could be an effective anti-inflammatory target for brain hemorrhage; PD-1 agonism was shown in an ICH model to reduce edema and improve neurological outcomes.^87^ Jackson et al. administered a PD-1 ligand to inhibit monocytes and showed significant reduction in ICA vasospasm 48 h after induction. There was no improvement in neurobehavioral performance at that time point, and the study did not persist into in the DCI time window.^88^ The authors propose that the attenuated vasospasm was due to decreased monocyte migration into the CNS, but the PD-1 pathway is known to affect neutrophil migration and activity as well which offers an additional possible mechanism.^89^

A primary role of neutrophils in the immune system is to act as first-responders in microbial defense. To accomplish this, neutrophils release their intracellular pool of granules, which contains many proteins, including neutrophil elastase and MPO. Another molecule utilized by neutrophils to combat microbes is neutrophil cytosolic factor 1 (NCF1), which is part of the complex responsible for producing reactive oxygen species. Since these are key proteins for neutrophils to perform their functions, Coulibaly et al. conducted a study to determine if any of these proteins are deleterious following SAH using transgenic mice deficient in one of these factors.^90^ Their study showed that MPO knockout protected SAH mice from delayed spatial memory deficits and MCA vasospasm, and also reduced neutrophil recruitment to the venous sinuses and meninges 3 days after SAH. To further support the role of MPO in SAH outcome, they injected MPO into MPO knockout mice which recapitulated the memory deficits seen in the wild-type SAH group (i.e. intracisternal MPO reversed the protective effect of MPO knockout). While MPO knockout provided beneficial outcomes after SAH, neither the neutrophil elastase nor the NCF1 knockout mice had improved outcomes compared to the wild-type SAH mice.^90^

These studies further support the theory that neutrophils are a key mediator of poor outcome after SAH, however work remains to be done to establish the pathophysiological events that these various therapeutic targets cause. For example, how is MPO causing MCA vasospasm and poor neurobehavior after SAH? Moreover, clinically translatable drugs do not exist for many of these targets which would be required for moving forward with a Phase 1 clinical trial.

### Cell adhesion molecules (CAM) and neutrophil migration

The role of CAMs in SAH were among the first series of experiments performed which suggested neutrophils are a therapeutic target for SAH since blocking CAMs attenuates neutrophil adhesion and migration out of the vasculature and into the CNS.^91,92^ In 1998, Bavbek et al. published the first study examining the effects of inhibiting ICAM-1 and CD18 using a rabbit SAH model. Treatment with antibodies for either ICAM-1 or CD18, two molecules known to be involved in leukocyte migration, reduced the amount of basilar artery constriction 48 h after SAH by 22% and 27%, respectively, and antibodies against both ICAM-1 and CD18 led to a 56% reduction in constriction.^93^ The findings for the CD18 antibody have been supported by two other studies. Pradilla et al., using a rabbit SAH model, reported that an anti-CD11/CD-18 antibody increased basilar artery luminal patency at 72 h after SAH to 90% (compared to 59% in the non-treated SAH group).^94^ Clatterbuck et al., also using an antibody against CD11 and CD18, reported that treatment increased the retained flow seen on PBD 7 angiograms to 95.8% compared to 53.0% in untreated SAH primates.^95^ In addition to CD18 and ICAM-1, two studies reported that inhibiting E-selectin, another CAM molecule associated with leukocyte migration, led to a significant reduction in arterial vasospasm after SAH in rodents.^96,97^

A caveat of ICAM-1, CD18, and E-selectin is that these CAMs are not specific to neutrophil migration, but rather contribute to CNS migration of several immune cell types. However, support for the theory that neutrophils are key to the pathophysiology of vasospasm and DCI after SAH is found in other mechanistic animal studies. One such study by Xu et al. utilized an endovascular puncture mouse SAH model to examine the effects of inhibiting VAP-1 (a protein expressed on the surface of endothelial cells, that is, important in the binding of leukocytes) and neutropenia on pial arterial dilation.^98^ Forty-eight hours after SAH induction, both VAP-1 antagonism and neutropenia preserved arterial response to vasodilator administration whereas the vehicles had a >80% drop in arteriolar diameter response. The authors also reported improved neurological outcome for VAP-1 inhibition and neutropenia.^99^ Regarding ICAM-1, Atangana et al. observed a significant reduction in neutrophil-endothelial interactions (rolling and adhering neutrophils) after SAH in ICAM-1 knockout mice and PSGL-1 knockout mice (PSGL-1 is a leukocyte receptor that interacts with ICAM-1). More interesting though is that Atangana et al. showed that the reduction in neutrophil extravasation significantly reduced microglia activation and cell death after SAH.^75^ This further suggests that neutrophils are involved in causing other deleterious events after SAH.

### NETs formation and intravascular occlusion of brain blood vessels

While the initial work involved in the discovery and function of NETs established their crucial role in bacterial infections, NETs are now known to also cause deleterious events following sterile injuries, including ischemic and hemorrhagic stroke,^100,101^ which may be via promoting thrombosis^102,103^ or microvascular dysfunction.^104,105^ Since NETs are deleterious in several pathologies, two general therapeutic strategies have been employed: inhibition of neutrophil NETosis (preventing the release and formation of NETs) and NETs degradation (after NETs have formed). While several key molecules have been identified as therapeutic targets to prevent NETosis (e.g. TLR4, PAD4), future studies examining the upstream signaling pathways of NETosis may identify more targets. Regarding NETs degradation, the most common methods is by using either DNase or RNase as both enzymes have been shown to break down extracellular DNA which is a key component of the NETs structure.^55^

To determine if NETs are pathological, four studies utilized neutrophil depletion as a method to reduce NETs. Zeng et al. and Hao et al. showed that neutrophil depletion reduced NET formation and improved neurological outcomes at 24 h, suggesting a reduction in EBI but did not study later time points.^68,83^ Work from our lab reports that mice depleted of neutrophils had significantly less intravascular NETs compared to non-depleted SAH mice on days 1 and 7. More interesting is that there was a positive correlation between intravascular NETs count and DND/DCI, and neutrophil depletion significantly reduced the incidence of DND/DCI.^71^ While our lab studied intravascular NETs, a recent study by Nakagawa et al. used neutrophil depletion to evaluate NETs in the perivascular space. They found that by PBD 1 neutrophils had migrated out of the pial arterioles into the perivascular space and began forming NETs. In their cohort of neutrophil depleted mice this migration and NET formation did not happen and that microvasospasms of the pial arterioles as seen on intravital microscopy was significantly reduced. Importantly, in their cisternal injection SAH model, Nakagawa et al. showed that the perivascular erythrocytes were from the injected blood as expected, but the perivascular neutrophils were almost entirely derived from the host animal’s intravascular blood.^106^

Four animal models of SAH have been utilized to understand the spatial and temporal patterns of NETs after SAH. Früh et al. observed that NETs first accumulate within the subarachnoid space after SAH, peaking on PBD 1 and returning to levels indistinguishable from sham by PBD 7. Regarding the brain parenchyma, NETs gradually increased overtime starting primarily in the basal ipsilateral hemisphere and spreading cortically and to the contralateral hemisphere through PBD 14 (the last time point in the study).^65^ Zeineddine et al. observed intravascular NETs in the brain peaked on PBD 1 with a second peak on PBD 7 (the last time point studied).^71^ Zeng et al. also found that NET formation had a peak at PBD 1, however the authors only analyzed NETs for up to 48 h.^68^ Lastly, Nakagawa et al. showed that NET formation takes place in the pial arterioles perivascular space as early as PBD 1, but this was only time period evaluated.^106^ Taken together, these studies highlight that NETs accumulate in the brain after SAH, there may be delayed NETs formation, and NETs may be pathological, especially with respect to DCI.

Since mechanisms causing NETosis are still being identified, most studies on NETs in SAH have focused on degrading NETs. To this end, both DNase I and RNase have been examined, with DNase I being more heavily tested, and treatment generally started within 3 h post-SAH. The studies agree that DNase I (or RNase) is capable of degrading NETs,^65,68,71,83,107^ with improved PBD 1 neurobehavioral outcomes.^68,83,107^ Other notable beneficial outcomes reported for DNase I treatment following SAH include reducing the levels of pro-inflammatory microglia and cytokines,^107^ less brain edema,^68,83,107^ reduced neuronal damage,^68,83,107^ and improved cortical perfusion at 6 h post-SAH.^83^ The study by Hao et al. examined CSF flow disruption 24 h after SAH, and found that DNase I treatment led to greater dye spread on the ventral brain surface and increased dye concentration in the deep cervical lymph nodes, suggesting improved CSF flow and absorption following DNase I treatment.^83^ Despite DNase I affording benefit to SAH mice during the EBI period, there may be less benefit for DNAse I with respect to DCI.

Only two studies examined the effect of DNase I treatment during the DCI time window, Zeineddine et al. and Nakagawa et al. Similar to others, Zeineddine et al. observed benefit by the DNase I treatment on PBD 1, and also reported reduced intravascular NETs on PBD 7. However, Zeineddine et al. found that despite reducing intravascular NETs, there was no reduction in infarct volume or DND compared to the vehicle group.^71^ It should be noted that all of the studies only administered DNase I one time on the day of SAH, so there is a chance that daily DNase I treatment may prevent DCI. As a single injection of DNase I can reduce PBD 1 NETs and prevent neurobehavioral deficits but does not reduce DCI incidence, perhaps it is the second peak of NETs around day 7, that is, the cause of DCI. Consistent with Zeineddine et al., Nakagawa et al. showed no reduction in microvasospasms with treatment of intraperitoneal DNase I 1 and 8 h after SAH . Despite a lack of treatment benefit by intraperitoneal injection, intracisternal DNase I administration on PBD 1 significantly reduced peri-vascular NETs within minutes after DNase I administration which corresponded with a reduction in microvasospasms on PBD 5.^106^ Neurobehavioral testing was not done in this study, but these results suggest that there may be important clinical differences in NETs location and as that the route and timing of therapeutics to degrade NETs is crucial.

NETs are reported to propagate several other deleterious pathophysiological events, including immune cell activation and inflammation, and platelet activation and microthrombi. As such, inhibiting NETosis, which is expected to prevent the formation of NETs altogether, may also minimize the cycle of thromboinflammation. Thus far, only two studies have examined NETosis inhibition, both targeting PAD4. During the EBI period, Zeng et al. found that pre-treatment with a PAD4 inhibitor significantly reduced NETs formation in the brain which corresponded with less brain edema and neuronal damage, leading to improved neurological function at 24 h. Interestingly, pre-treatment with the PAD4 inhibitor was also capable of improving long-term cognitive function, 28 days after SAH.^68^ The beneficial effect of PAD4 inhibition for SAH has also been supported by the findings of Zeineddine et al. Specifically, PAD4 inhibition 1 h post-SAH reduced the formation of intravascular NETs and improved PBD 1 neurological function. Regarding the DCI period, Zeineddine et al. reported that post-SAH PAD4 inhibition led to reduced delayed infarct volume and DND.^71^ Together, these reports suggest that inhibiting PAD4 may be a therapeutic target to improve outcome for SAH patients, but, more importantly, these studies indicate that NETs are deleterious and they need to be targeted by treatments. It should also be noted that PAD4 inhibition seems to be more beneficial than degrading NETs since PAD4 inhibition prevented DCI but DNase I did not.^71^ While the reason why prevention of NETosis is superior to NET degradation has not been examined, it may be due to the prevention of a deleterious ischemic, vascular occlusion, or inflammatory cascade that has already taken effect by the time DNase begins its NET degradation. Vascular occlusion by NETs may cause localized damage before NET degradation. NETs are also known to promote coagulation, platelet activation, and inflammation. Thus, preventing NETs may reduce these deleterious events while degrading NETs may allow these events to initiate. Additionally, PAD4 is known to be involved in neutrophil migration, the inhibition of which could lead to reduced DND in a similar manner as neutrophil depletion.^108,109^ Other pleotropic effects of PAD4 inhibition or off-target effects of the specific PAD4 inhibitors are possible explanations as well, but were not evaluated in these studies.

A final study on NETs considered the hypothesis that TREM1 promotes NETs production through recruitment of spleen tyrosine kinase (SYK). TREM1 is part of a family of innate immune receptors that can imitate and enhance inflammation and is expressed on the surface of myeloid cells. It is known to act on the SYK pathway, which among other proinflammatory activities can activate PAD4 related NETosis. Wu et al. used an inhibitor of the TREM1 signaling pathway and showed that TREM1 inhibition reduced NETs formation and systemic inflammatory cytokines, improved neurobehavioral performance, prevented microglia activation, decreased brain edema, and lessened neuronal damage at 24 h compared to injured controls. On the other hand, administration of TREM1 worsened brain edema and neurobehavioral outcomes, but inhibition of SYK reversed the damaging effects of TREM1 administration.^67^ Importantly for the mechanistic understanding, the inhibition of both TREM1 and its downstream effector SYK showed decreased levels of PAD4 strongly suggesting a direct link between TREM1 and NETosis.

It remains unknown though if TREM1 directly induces NETs, but since TREM1 is known to synergistically amplify inflammation with toll-like receptor (TLR) signaling,^110^ and as TLRs, especially TLR4, are known to directly cause NETosis,^111^ TREM1 may be at the very least an accessory receptor to promote NETosis.

## Future directions

While the direct evidence for the role of neutrophils in neurological damage after SAH in animal models is substantial, progress in establishing a causative role in humans has been hindered by limitations on studying neutrophils in the brain after SAH. The paucity of clinical trials targeting neutrophils is presumably due to the potential detrimental side effects that neutrophil depletion would have on a population known to be vulnerable to infections.^84^ In order for neutrophils to be relevantly targeted by therapeutic interventions in humans, our understanding and identification of critical neutrophil mechanisms need to be advanced. In the last few years, rodent studies have suggested that NETs may offer some potential for clinical translation as NETs inhibition and NETs degradation may be less immunosuppressive than neutrophil depletion. To date, no human clinical trial has been completed examining either strategy.^112^

While the evidence for NETs is robust, other mechanisms by neutrophils may also play a critical role in contributing to DCI. Many components of neutrophil granules such as ROS, prostaglandins, and proteolytic enzymes are known to either damage the endothelium, or cause vasoconstriction; both of which may contribute to DCI.^113,114^ However, to date there have been no studies looking directly at this in animal SAH models. Indirectly though, some of the previously mentioned studies did use these granule components such as MPO to indicate neutrophil activity lending some plausibility to alternative mechanism.

## Conclusions

Neuroinflammation is known to be important in the pathogenesis of DCI and DND. Neutrophils are a key immune cell and early responder in the activation of the innate immune system and this paper summarizes the evidence of neutrophils’ roles in SAH for both humans and animal models. There is robust animal model data reporting the deleterious effects of neutrophils on SAH outcome and several studies have demonstrated the therapeutic potential of inhibiting neutrophil function. However, in humans the evidence is only indirect and no clinical trials targeting neutrophils have been initiated. The inhibition of NETosis or degrading NETs offer new therapeutic targets that have the potential for preventing DCI while maintaining a responsive immune system.
